# *In Vivo* Early Intervention and the Therapeutic Effects of 20(S)-Ginsenoside Rg3 on Hypertrophic Scar Formation

**DOI:** 10.1371/journal.pone.0113640

**Published:** 2014-12-12

**Authors:** Liying Cheng, Xiaoming Sun, Changmin Hu, Rong Jin, Baoshan Sun, Yaoming Shi, Wenguo Cui, Yuguang Zhang

**Affiliations:** 1 Department of Plastic and Reconstructive Surgery, Ninth People's Hospital affiliated to Medical School of Shanghai Jiao Tong University, 639 Zhi Zao Ju Road, Shanghai 200011, People's Republic of China; 2 Orthopedic Institute, Soochow University, 708 Renmin Road, Suzhou, Jiangsu 215006, People's Republic of China; University of Tennessee, United States of America

## Abstract

**Background:**

Intra-lesional injections of corticosteroids, interferon, and chemotherapeutic drugs are currently the most popular treatments of hypertrophic scar formation. However, these drugs can only be used after HS is formed, and not during the inflammatory phase of wound healing, which regulates the HS forming process.

**Objective:**

To investigate a new, effective, combining therapeutic and safe drug for early intervention and treatment for hypertrophic scars.

**Methods:**

Cell viability assay and flow cytometric analysis were studied *in vitro*. Animal studies were done to investigate the combining therapeutic effects of 20(S)-ginsenoside Rg3 (Rg3) on the inflammatory phase of wound healing and HS formation.

**Results:**

*In vitro* studies showed that Rg3 can inhibit HS fibroblasts proliferation and induce HSF apoptosis in a concentration-dependent manner. *In vivo* studies demonstrated that Rg3 can limit the exaggerated inflammation, and do not delay the wound healing process, which indicates that Rg3 could be used as an early intervention to reduce HS formation. Topical injection of 4 mg/mL Rg3 can reduce HS formation by 34%. Histological and molecular studies revealed that Rg3 injection inhibits fibroblasts proliferation thus reduced the accumulation of collagen fibers, and down-regulates VEGF expression in the HS tissue.

**Conclusion:**

Rg3 can be employed as an early intervention and a combining therapeutic drug to reduce inflammation and HS formation as well.

## Introduction

Hypertrophic scar (HS) formation is a fibrotic disease characterized by a raised scar (red or pink), which is sometimes pruritic but does not exceed the margins of the original wound. This process is distinguished by three distinct but overlapping phases: the inflammatory, proliferative and remodeling phases. According to current researches, exaggerated inflammation, augmented neovascularization, reduced apoptosis, overabundant extracellular matrix (ECM) deposition, atypical ECM remodeling are the main mechanisms of HS formation [1]. And targets of treating HS are always to inhibit these pathological mechanisms.

The main treatment for HS formation consists of intralesional injections of corticosteroids [2]–[4], interferon [5]–[9], and chemotherapeutic drugs [10]. Recent years, vitamin D derivative (20(OH) D3) has been found to have antifibrogenetic activity, and may be used for a treatment of HS [11]–[14]. Unfortunately they can only modify proliferation and remodeling once the HS has formed, and have no effect in the early inflammatory phase of wound healing. In addition, these therapies only worked in one or two mechanisms after HS formed, and have no effect on the exaggerated inflammation, or augmented neovascularization, which are two important mechanisms of HS formation, and therefore the treatment results are compromised. Moreover, corticosteroids, 5-Fu (5-Fluorouracil) and interferon injection may cause wound dehiscence, pupura and other undesirable complications [15]. Therefore, such treatment is far from a perfect regime for HS patients.

Early intervention and combining two or more therapeutic targets hold great potential to revolutionize HS therapy. The ideal drug therapy for HS should target the inflammatory phase and limit the exaggerated inflammation, promote wound healing, and thus shorten the reepithelialization time. This is of highly importance since scar hypertrophy is more likely to occur if wound closure requires more than three weeks [16]. Meanwhile, such HS therapies should target the many mechanisms of HS's formation. Multi-target therapy has a better potential to improve the prognosis of HS than traditional single target therapy.

20(S)-ginsenoside Rg3 (Rg3), one of the main constituents found in red ginseng, has been used as folk medicine for thousands of years in China, and has been shown to have a wide spectrum of pharmacological effects. It can inhibit the proliferation of various kinds of cancer cells, and induce their apoptosis [17]–[20]. Recently, Rg3 was found to be capable of inducing the apoptosis of human lung fibroblasts [21]–[22]. Some reports have indicated that it can suppress the production of some proinflammatory cytokines [23]–[24]. In addition, studies have shown that Rg3 can inhibit exenograft growth and angiogenesis in tumors primarily via the down-regulation of VEGF expression [25]–[26], and can inhibit the proliferation of human umbilical vein endothelial cells [27]. Above all of this, Rg3 is a Chinese herb which has been shown to be safe and free of side effects [28]–[30].

Given the above characteristics of Rg3, we hypothesized that Rg3 would limit the exaggerated inflammation in the proliferative phase, inhibit fibroblast proliferation and augmented neovascularization in the remodeling phase. The early intervention and the combined therapeutic function targeting 3 mechanisms together may shed new light on HS treatment and lead to an obviously reduction in HS formation, which may be more effective than traditional drug injection.

In this study, we sought to investigate the inhibitory effect of Rg3 on the *in vitro* proliferation of human HSFs. Further, we generated the rabbit ear HS model to investigate whether intralesional injections of Rg3 beginning in the early phase of wound healing could prevent or reduce *in vivo* HS formation.

## Materials and Methods

### Chemicals

Ginsenoside Rg3 (Rg3) is a reference compound (purity >99.5%) purchased from Fusheng Pharmaceuticals Inc. (Dalian, China). Rg3 was dissolved in dimethyl sulfoxide (DMSO) and filtered by 0.2 µm membrane. The final concentrations of DMSO in the culture medium were <0.05%. It was diluted by cell culture media to various final concentrations (0, 25, 50, 75, 100, 200 µg/mL) for the *in vitro* study, and diluted by saline to various final concentrations (1, 2, 3 and 4 mg/mL) for the *in vivo* study.

The concentrations of the Rg3 were designed according to references [30]–[33]. The dosages used in the present study were based on existing data of the effective dose, and the results of an acute toxicity study of Rg3 on mice [20]–[34]. In the pharmacodynamic test, the therapeutic dose was 1 mg/kg. In the acute toxicity study, the LD50 was considered to be more than 60 mg/kg with intraperitoneal injection, and the maximum tolerance dose was considered to be more than 90 mg/kg with intramuscular injection. A dose less than 90 mg/kg is the safety range of Rg3 for injection. In many studies, Rg3 was used by intraperioneal injections with a concentration of 3.0–10.0 mg/kg. In our present study, 4.0 mg/mLwas specified as the highest dose level, which was equal to 4.8 mg/kg, with lower doses being 1.2, 2.4, 3.6 mg/kg, and these were respectively 1.2, 2.4 and 3.6 times the therapeutic dosage.

### Cell culture

HS tissue was obtained from patients (6 male and 6 female) by intramarginal excision of the lesions according to standard surgical procedures in the Department of Plastic and Reconstructive Surgery, Shanghai Ninth People's Hospital affiliated to Shanghai Jiao Tong University, School of Medicine. Only virgin mature pathologic scars without prior steroid injection, radiation therapy, and surgical excision were selected. Before surgery, all the patients were informed of the purpose and procedure of this study and agreed to offer their excessive tissue. The written consent was obtained from all participants involved in this study. All the protocols were approved by the Ethic Committee of Shanghai Ninth Peolple's Hospital affiliated to Shanghai Jiao Tong University, School of Medicine.

HSFs were isolated by sequential trypsin and collagenase digestion. HSFs were cultured in high glucose Dulbecco's modified Eagle medium (DMEM) supplemented with 10% fetal bovine serum (FBS, Sijiqin Co. Hangzhou, China), penicillin (100 units/mL) and streptomycin (100 µg/mL) (Sigma) in the 100 mm diameter ×15 mm height dishes (Thermo Scientific) in a humidified incubator at 37°C with 5% CO_2_. Culture media were replaced every three days. Once adequate growth of fibroblasts was detected, the fibroblasts were trypsinized for subculture in 10% FBS–DMEM. Only the second to fourth passages of HSFs were used in this study.

### Cell viability assay

The viability of HSFs treated with and without Rg3 was determined by CCK-8 assay (Cell counting kit-8, Dojindo, Kumamoto, Japan). Briefly, HSFs were seeded in 96-well plates (Thermo Scientific) at a density of 1×10^4^ cells/well and allowed to adhere for 24 h at 37°C under 5% CO_2_. After incubation, Rg3 was added to the medium to different final concentrations: 0, 25, 50, 75, 100 and 200 µg/mL. On day 1, 2, 3, 4, and 5 after incubation, 15 µL CCK-8 was added to each well, and incubated at 37°C for 2.5 h according to the reagent manufacture's instruction. 100 µL aliquot of incubated medium was pipetted into a 96-well plate, and a microplate reader (Thermo labsystems, Helsinki, Finland) was used to detect the absorbance of each well at 450 nm. To determine the percentage of surviving cells, absorbance values of indicated concentrations were normalized to the values obtained from the cells without Rg3 treatment. Each assay was performed in 3 replicates.

### Microscopic observation

To assess the cell death induced by Rg3, we analyzed morphological changes. HSFs were seeded in 6-well plates (Thermo Scientific) at 5×10^4^ cells/well and cultured with 5% CO_2_ at a temperature of 37°C. HSFs were subsequently divided into six groups (1 control groups and 5 experimental groups) with different concentrations of Rg3: 0, 25, 50, 75, 100 and 200 µg/mL. Following a 48 h incubation period, the HSFs in different groups were viewed and images were obtained using an inverted microscope (Nikon IX70, Japan). Experiments were performed in triplicate.

### Flow cytometric (FCM) analysis of annexin V-FITC staining

The rate of apoptosis was measured by FCM according to the instructions provided by the annexin V-FITC kit. In brief, The HSFs were incubated on the 6-well plates in medium with 0, 25, 50, 75, 100, 200 µg/mL Rg3. After 48 h of incubation, the HSFs were collected by centrifugation, washed twice with cold PBS, and resuspended in binding buffer at a concentration of 1×10^6^ cells/mL, in which 100 µL of cell suspension was added to a 5 mL FCM tube. A total of 5 mL of annexin V-FITC and 1 ml of 20 mg/mL propidium iodide (PI) were added and incubated for 15 min at room temperature in the dark before a further addition of 400 µL of PBS. Quantitative analysis of apoptotic levels was performed using a flow cytometer (BD Bioscience). The apoptotic percentage of 10000 cells was determined and all the experiments reported in this study were performed in triplicate. Data analysis was performed using Cell Quest software (BD Bioscience).

### Rabbit-ear HS model

The rabbit ear wound healing and HS model was used as described previously with minor modifications [35]. Eighteen New Zealand White rabbits weighing 3.5±0.5 kg were purchased from the Shanghai Animal Center at the Chinese Academy of Science. The Institutional Review Committee of Shanghai Jiao Tong University, School of Medicine approved all animal study protocols, and the experiments were carried out strictly with the approval of the Animal Experimentation Ethics Committee of Shanghai Jiao Tong University, School of Medicine. (Animal care and use committee of Shanghai Jiao Tong University, certificate number: SYXK [Hu] 2012-0007)

For the surgical procedure, animals were anesthetized using an intramuscular injection of ketamine (10 mg/kg) and xylazine (3 mg/kg). Carefully avoiding the central ear artery and marginal ear veins, six full-thickness wounds (down to the cartilage) were created on the ventral side of each ear using a 10-mm punch biopsy tool. The epidermis, dermis and perichondrium on the cartilage were thoroughly removed. Each two wounds were separated by at least 10 mm. Occasional bleeding was treated using manual compression. Totally there were 216 wounds created on 18 rabbits.

### Treatment and grouping

The rabbits were randomly divided into six groups, with 12 scars in each group: one control group, one saline-treated (placebo) group and four Rg3-treated groups. Pharmacokinetic studies suggested that ginsenosides are very poorly absorbed following oral administration to rabbits [36]. so here we use Rg3 injection, and the concentration of Rg3 was set at 1, 2, 3 and 4 mg/mL according to previous study [30]–[34], [37]. The wounds in the control group were untreated. The wounds in the saline-treated (placebo) group were injected with saline instantly after operation and every three days a time thereafter. Rg3 (1, 2, 3 and 4 mg/mL) was applied instantly after operation and three days a time to the wounds in the four treatment groups. Wounds were observed daily and photographs were taken on postoperative Day 28. Animals were sacrificed on postoperative Day 28 and scars were harvested.

### Histologic analysis

All the tissue samples were cut in a full-thickness manner from the wound site and bisected at the maximum point of scar hypertrophy (judged visually and by palpation). One-half of the harvested samples were fixed in 4% formaldehyde/PBS solution at 4°C, dehydrated in a graded series of ethanol and then embedded in paraffin for histological analysis. The samples were sectioned at 5 µm, stained using hematoxylin and eosin (HE) and visualized using an optical microscope. The other half samples were stored at −80°C for RNA extraction and VEGF quantification.

### Analysis of inflammatory cells in the wound tissue on day 14

HE staining was performed in the wound tissue on day 14. All the tissue sections were observed under a microscope at 100× and 400× magnification respectively, and the images were digitally recorded into a computer by the Image-Pro Plus system. Inflammatory cells (lymphocytes, heterophils (rabbits' nutrophils), and macrophages) were identified by characteristic morphology [38]–[39]. The number of inflammatory cells in the scar tissue on day 14 in five random high-power (400×) fields from each section was counted. Only intact inflammatory cells with distinct blue staining were counted.

### Scar elevation index

The extent of scar hypertrophy was expressed as the scar elevation index (SEI). The SEI measures the ratio of total scar area to the area of normal underlying dermis, as described previously by Morris et al [35], [37], [40], [41]. The SEI of both treated and untreated wounds was measured. The height of the underlying dermis was determined based on the height of the adjacent unwounded dermis. Using the HE-stained tissue sections, measurements were taken within the wounded area at 100× magnification. The epithelial height was not considered for SEI calculations. SEI integrates cell proliferation, dermis thickness, and matrix deposition as an area under the curve, and this measurement correlates highly with other measurements of wound healing. An SEI of 1 indicates no newly formed hypertrophied dermis, whereas an index >1 denotes HS formation. The SEI was measured twice by a blinded examiner, and the two values were then averaged.

### Epidermal thickness index

The epidermal thickness index (ETI) was utilized to determine the degree of epidermal hypertrophy and was based on measurements taken from HE-stained tissue sections at 400× magnification. The entire cross section of the scar was measured for epidermal thickness, which corresponded to a total of approximately 5 fields. The epidermal thickness from 5 fields of uninjured skin was also measured from both sides of the scar. The ETI was then determined by calculating the ratio between the averaged epidermal height in scar tissue and the averaged epidermal height in normal uninjured skin [35]. An ETI >1 denotes hypertrophic epidermis forming.

### Masson's trichrome staining for collagen fibers

Paraffin-embedded sections (5 µm) were mounted on glass slides and stained for collagen using Masson's Trichrome (collagen stains green). For each wound, 5 randomly chosen fields of dermis were photographed at 400× magnification, and density of collagen fibers was detected.

### Immunohistochemical analysis of Col-I

Immunohistochemistry was performed on 5-µm-thick paraffin-embedded tissue sections. The sections were dewaxed, and endogenous peroxidase activity was quenched with 3% hydrogen peroxide for 10 min, followed by blocking with the corresponding serum from a secondary antibody raised animal species for 1 hour. Slides were then incubated overnight at 4°C with the primary antibody mouse anti-Col-I antibody (1∶1000, abcam, USA) diluted in the blocking serum. After several washes with PBS, the tissue sections were incubated with peroxidase-conjugated goat anti-mouse polyclonal antibody (DAKO, Carpinteria, CA) at a dilution of 1∶200 in PBS for 30 min at 37°C. After several washes in PBS, the signals on the tissue were revealed by incubating with DAB in PBS for 5–10 min, followed by hematoxylin counterstaining. All the tissue sections were observed under a microscope (Nikon, Japan) at a 400× magnification, and 5 randomly chosen fields of dermis were photographed for each wound. The sizes of areas of collagen deposition were estimated using the Image Pro Plus 6.0 software. The relative density of collagen in each group was calculated by normalizing to the unwounded skin collagen density.

### VEGF expression analysis by reverse transcription-polymerase chain reaction (RT-PCR)

For RT-PCR, −80°C frozen samples (50 mg) were crushed in 1 ml Trizol reagent (Invitrogen, US) with the homogenizer according to the manufacturer's recommendations. RNA concentrations were measured using the NanoDrop spectrophotometer (NanoDrop Technologies, Wilmington, DE). Equal concentrations of RNA samples (2.0 µg) were then reverse transcribed to cDNA by the Thermo Script RT-PCR system (Gibco-Invitrogen Corporation, Burlington, ON, Canada) following the manufacturer's protocol.

The real-time RT-PCR reactions (20 µL) consisted of 10 µL PCR Premix Mix (1×) (TaKaRa, Dalian, China), 2.0 µL PCR forward primer (0.2 µM), 2.0 µL PCR reverse primer (0.2 µM), 1 µL cDNA and 5 µL double-distilled water. Primer pairs were as follows:

- 5′-ACGAAGTGGTGAAGTTCATGGAA-3′ (forward);
- 5′-AAGATGTCCACCAAGGTCTCGAT-3′ (reverse).

The gene of interest was normalized against the reference gene glyceraldehyde-3-phosphate dehydrogenase (GAPDH):

- 5′-ACTTTGTGAAGCTCATTTCCTGGTA-3′ (forward);
- 5′-GTGGTTTGAGGGCTCTTACTCCTT-3′ (reverse).

A typical protocol included initial denaturation at 95°C for 30 s, followed by 40 cycles with denaturation at 95°C for 5 s, annealing at 60°C for 30 s, followed by a melt curve 15 s at 95°C ramp to 60°C for 10 s, followed by 15 s at 95°C. PCR was performed in triplicate. The expression level of each target gene was calculated as 2^−ΔΔCt^.

### Statistical analysis

All experiments were performed in triplicate, and each datum was calculated from replicates of three. Data are expressed as the mean ± standard deviation (SD). Statistically significant differences (*p<0.05*) among the various groups were measured using two-tailed Student's t-test. All statistical analysis was carried out using the SAS 8.2 statistical software. A probability value (p) of less than 0.05 is considered as statistically significance.

## Results

### Rg3 represses HSFs viability in a concentration-dependent way

**Figure 1:**
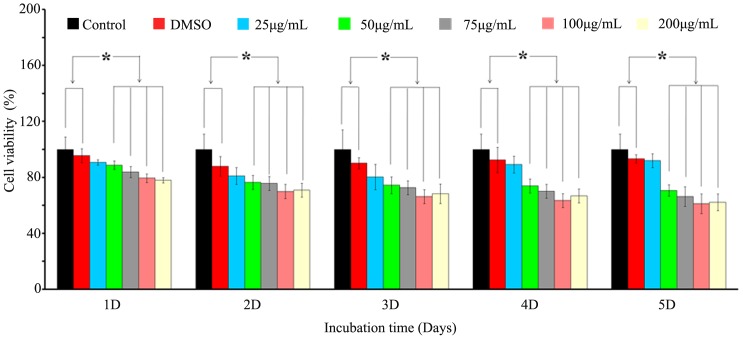
The effect of 20(S)-Rg3 on hypertrophic scar fibroblast viability. Data are expressed as the mean ± SD of triplicate experiments. * *p<0.05* is considered to be significantly different compared to the control, as determined by the two-tailed Student's t-test.

As shown in Fig. 1, when HSFs were treated by 25 µg/mL Rg3, the HSFs viability was not significantly decreased. However, when HSFs were treated by 50, 75, 100 and 200 µg/mL Rg3, the cell viability significantly decreased (*p<0.05* vs control). The viable HSFs consistently decreased as the concentrations of Rg3 increased. However, when the Rg3 concentration was 200 µg/mL, the inhibition rate rose in 50, 75 and 100 µg/mL Rg3 then declined slightly. The inhibition rate in the 100 µg/mL Rg3 group was the highest in the five concentrations. Therefore, 100 µg/mL Rg3 is the best concentration for inhibiting HSFs proliferation.

The cell viability increased in the control group, the 25, 50 µg/mL Rg3 groups, while decreased in the 75, 100 and 200 µg/mL Rg3 groups from day 1 to day 5.

### Rg3 induced HSFs morphological changes *in vitro*

**Figure 2:**
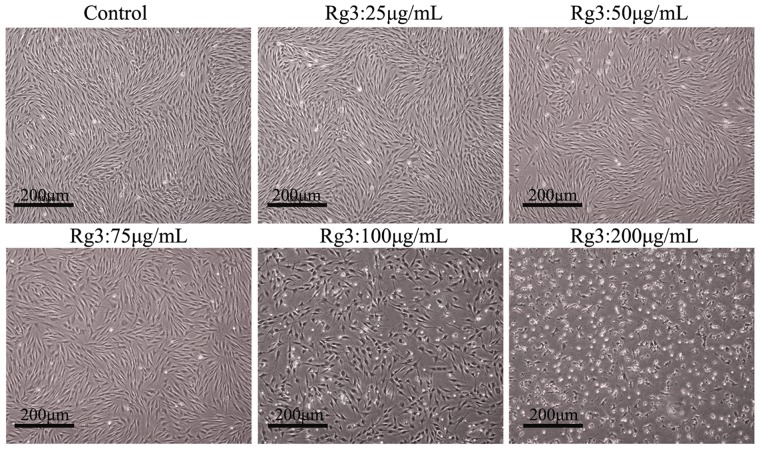
Morphology of HSFs treated with different concentrations of Rg3. Adherent HSFs were untreated or treated with 25, 50, 75, 100, 200 µg/mL Rg3 for 48 h. Images were taken using a light microscope and a digital camera. Original magnification: 40×.

We investigated the effect of different concentrations of Rg3 (0, 25, 50, 75, 100, 200 µg/mL) on HSFs morphology by microscopy for 48 h culture (Fig. 2). As is shown in Fig. 2, the density of HSFs gradually decreased as the concentrations of Rg3 increased. Treatment with 25, 50, and 75 µg/mL Rg3 for 48 h did not affect HSFs morphology, whereas treatment with 100 and 200 µg/mL Rg3 displayed typical apoptotic morphology, which showed round and oval-shaped cells, reduced volume, scattered between cell mass, condensed chromatin, and in a poor state, compared to the control cells without Rg3 treatment (Fig. 2). This result indicated that Rg3 could effectively inhibited HSFs proliferation in a concentration-dependent manner.

### Flow cytometric (FCM) analysis of HSFs apoptosis

**Figure 3:**
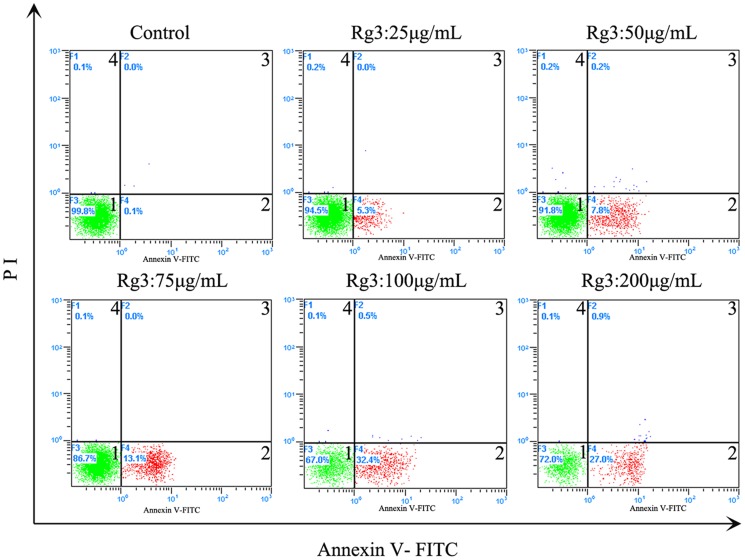
Effect of different concentrations of Rg3 on HSF apoptosis 48 h after treatment. Percentage of apoptotic cells was measured by flow cytometry after annexin-V/propidium iodide (PI) staining. Quandrant 1 indicates live cells; quadrant2 indicates cells in early apoptosis; quadrant 3 indicates cells in late apoptosis; quadrant 4 indicates cells in necrosis.

The number of apoptotic cells increased, as the concentration of Rg3 increased. The corresponding quantities of total cell apoptosis were 5.3±0.61, 9.2±0.5, 15.9±0.4, 36.6±0.4 and 40±0.6% at 25, 50, 75, 100, 200 µg/mL Rg3 (Fig. 3), respectively. Most of the apoptosis of HSFs in each group proved to be early apoptosis, and only a very small percentage stained positive for PI, indicating that the cells were not necrotic (data not shown). The above results indicated that high doses of Rg3 induced HSFs apoptosis in a concentration-dependent way, and that Rg3 is a safe drug with low cell-toxicity which won't cause cell necrosis.

### *In vivo* anti-inflammation effect and wound healing observation

The wound healing time in the untreated group (A) and the saline treated group were 18.75±1.42 d and 18.67±1.23 d, respectively, which were not significantly different. The wound healing time in the Rg3 treated groups (1, 2, 3 and 4 mg/mL) were 18.17±1.64, 18.5±1.45, 17.75±1.60 and 18.25±1.42 d, respectively, which were not significantly different compared with the untreated group and the saline treated group.

**Figure 4:**
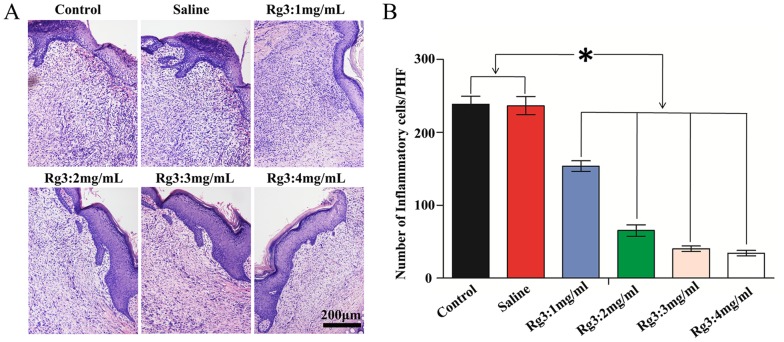
Histological observation of inflammation on day 14. (A) The inflammatory cells, granular tissue and newly formed epithelium could be observed by HE staining in each group. The Rg3 treated groups, the inflammation significantly decreased, with fewer inflammatory cells and newly-formed granular tissues density, compared with the untreated control group and the saline treated group. (HE, 100×) (B) Quantification of inflammatory cells in each group. The number of inflammatory cells in the Rg3 treated groups is lower than the control group and the saline injection group. (* indicates significant difference, p<0.05, data represent the mean ± SD of n = 9.)

HE staining of histological sections of all the experimental groups exhibited abundant inflammatory cells, newly-formed granulation tissues, and newly formed epithelium on postoperation day 14. (Fig. 4, A) In the untreated control group and the saline treated group, severe inflammation was observed at the repair site with numerous inflammatory cells, granular tissues, and newly-formed thickened epithelium. In the Rg3 treated groups, the inflammation reaction was also observed, but with a significantly decrease of inflammatory cells and newly-formed granulation tissues density.

Fig. 4 B, shows that there are much more inflammatory cells infiltrating the scars by week 2 in the control and the saline injection groups. Quantification of inflammatory cell infiltration in week 2 are 4.6±1.7, 8.0±2.3, 9.33±1.4, 12.0±3 PHF (Per high-power microscopic field), respectively. The number of inflammatory cells in the Rg3 treated groups is lower than that of the control group and that of the saline injection group. This anti-inflammatory effect was enhanced as the concentration of Rg3 increased. These results imply that injection of Rg3 could inhibit exaggerated inflammation to a certain extent in a dose-dependent mode, but won't affect the wound healing process, which might be very beneficial for anti-scarring therapy.

### Macroscopic evaluation of hypertrophic scarring

**Figure 5:**
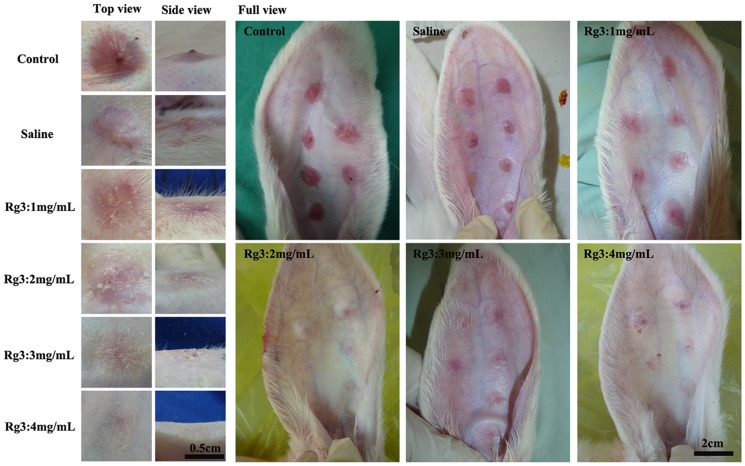
The clinical appearance of the wounds and scars. The top views, side views and full views of the scars that were left untreated and those that had been treated with different concentrations of Rg3 (0, 1, 2, 3 and 4 mg/mL). The wounds that had received injections of Rg3 (1, 2, 3 and 4 mg/mL) are obviously flattened and less reddish than the control and the placebo groups.

As shown in Fig. 5, on day 28, the control group and the saline treated group showed a visibly raised, contracted, reddish and palpable scar with evidence of HS. The wounds treated with different concentrations of Rg3 (1, 2, 3 and 4 mg/mL) also showed some evidence of HS, but the scars were less contracted, less raised and less reddish. The scars turned flatter as the concentration of Rg3 increased, and they are the flattest in the 3 mg/mL and 4 mg/mL Rg3-treated groups.

### Changes in histologic characteristics

**Figure 6:**
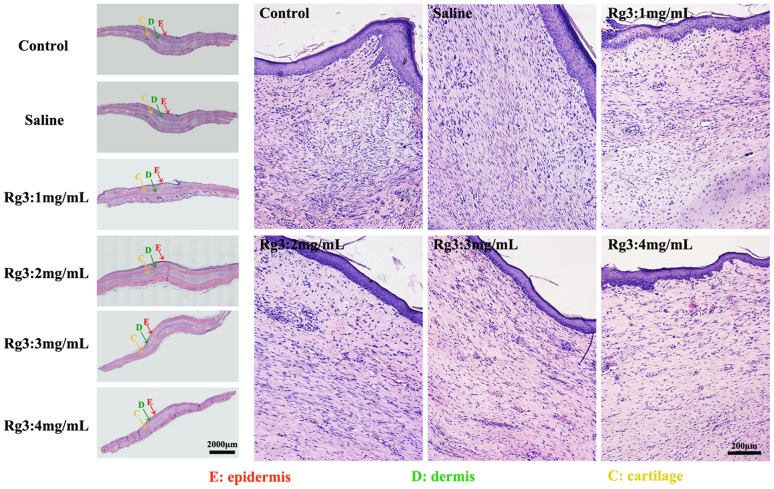
H&E staining of scar hyperplasia on day 28 after wounding. The 1^st^ column shows H&E staining at 40×. The 2–4^th^ columns show H&E staining at 100×. The dermal layer of the untreated control scars and the saline-treated scars were much thicker than Rg3 (1, 2, 3 and 4 mg/mL)-injected groups. The dermal layer was the thinnest in the high concentration Rg3-treated groups (3 and 4 mg/mL).

Histological analysis was performed to assess the anti-scarring properties of the different concentrations of Rg3 (1, 2, 3 and 4 mg/mL) on full-thickness rabbit ear wound, and the results are presented in Fig. 6. On day 28, there were typical infiltrations of fibroblasts in all groups, with different distributions and density. In the untreated control scars and the saline-treated scars, the epidermis and the dermis layer were significantly thickened. Abundant fibroblasts and thick tight collagen fibers were observed in the whole dermal layer, with a badly organized arrangement. The boundary between the papillary and reticular layers of the dermis was obscure. In comparison, the fibroblasts were mostly distributed in the upper dermis layer in the Rg3 (1, 2, 3 and 4 mg/mL)-treated groups, and the collagen fibers were assembled in parallel. The basal layer of the epidermis was flattened, and the thickness of the epidermis and the dermis layer was decreased. The density of fibroblasts, and collagen fibers were all reduced in the Rg3 (1, 2, 3 and 4 mg/mL)-treated groups, and the reduction is most obvious in the high concentration Rg3-treated groups (3 and 4 mg/mL). Quantification of the thickness of dermis layer is shown in the following subsection.

### Scar Elevation Index

**Figure 7:**
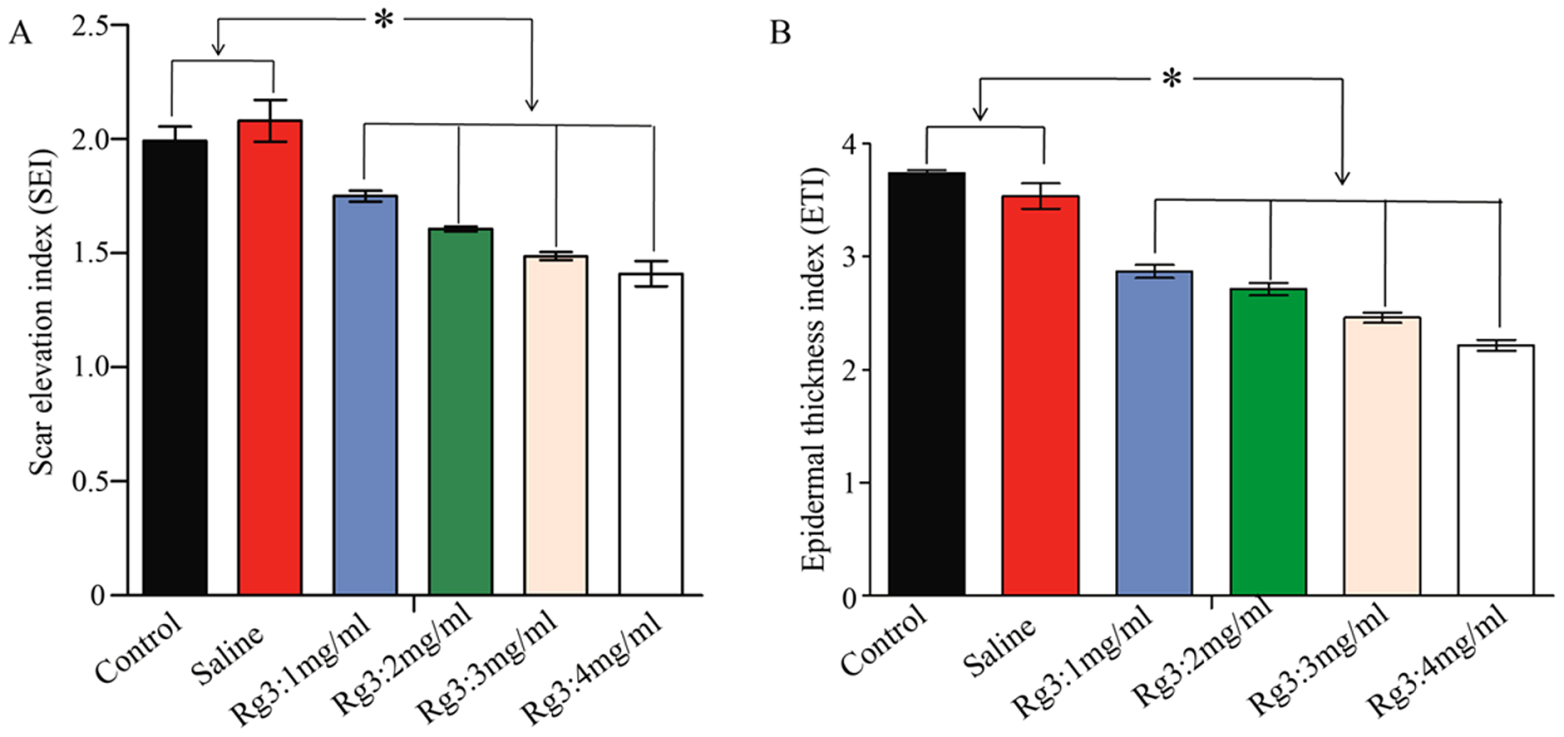
Scar Elevation Index (SEI) and Epidermal Thickness Index (ETI). (A) Dermal hypertrophy was measured by SEI. SEI in the Rg3 injection groups was compared with the control group and the saline injection group. SEI 1 depicts a hypertrophic scar. (B) Epidermis thickness was measured by ETI. ETI in the Rg3 injection groups was compared with the control group and the saline injection group. ETI >1 depicts a hypertrophic epidermis. (* indicates significant difference, p<0.05, data represent the mean ± SD of n = 9 wounds.)

To quantify the degree of scar formation, SEI was measured for nine different wounds for each treatment. The mean SEI in the untreated wounds and the saline-treated wounds were 2.29±0.10, 2.19±0.17, which were much higher compared with the SEI for wounds treated with different concentrations of 1, 2, 3 and 4 mg/mL Rg3 for 1.88±0.08, 1.70±0.08, 1.60±0.05 and 1.51±0.06, (n = 9, *p<0.01*), respectively. (Fig. 7, A) These data reveal a significant reduction in HS of 17, 25, 30 and 34%, respectively. Collectively, these data indicate that Rg3 injections are capable of reducing HS formation and inhibiting HS proliferation in a concentration-dependent mode.

### Epidermis thickness and epidermal thickness index

The integrity and thickness of epidermis is a feature of HS. As could be observed from fig. 6, samples from the untreated group and the saline injection group showed a visibly thicker epidermis than samples from the Rg3 injection groups (1, 2, 3, 4 mg/mL) on day 28. Notably, the epidermis was the thinnest in the Rg3: 4 mg/mL group.

To quantify the differences in epidermal thicknesses, ETI was measured in nine different wounds. Epidermal thickness was reduced in wounds treated with Rg3 injection to a mean ETI of 2.87±0.06 (1 mg/mL), 2.71±0.06 (2 mg/mL), 2.46±0.08 (3 mg/mL), and 2.22±0.05 (4 mg/mL) compared with untreated controls (3.74±0.05) at 1 month (Fig. 7, B). This reduction corresponds to a 23%, 28%, 34%, and 41% reduction (p<0.01) in ETI, respectively. Taken together, these results showed that the epidermal thickness decreased significantly in HS treated with Rg3 injection.

### Collagen density

**Figure 8:**
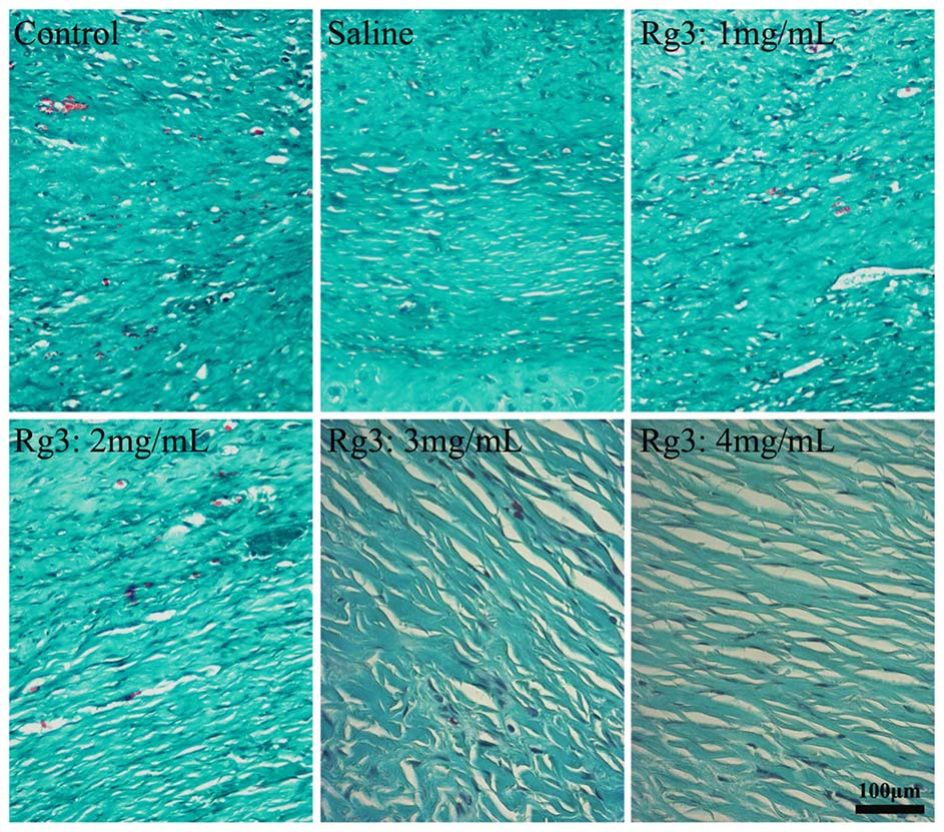
Masson's trichrome analysis of collagen accumulation. In the control group and the saline-treated group, the collagen fibers are thick, dense and disorganized. In tissues that had been treated with Rg3 (1, 2, 3 and 4 mg/mL) for 28 days, the collagen fibers were arranged more regularly and were more sparse than those in the control group. Original magnification: 400×.

Atypical ECM remodeling is another characteristic of HS, which is exhibited by overproduction of collagen. Masson's trichrome staining of the HS tissues was carried out. Light microscopic examination revealed typical features of collagen fibers in the HS tissue (Fig. 8) in the control group and the saline-treated group, compared with unwounded normal dermal tissue. The collagen bundles were thicker, denser, more disorganized and more abundant. By contrast, collage fibers were thinner, less dense and more regularly ranged in the Rg3 (1, 2, 3 and 4 mg/mL)-treated groups, which found dose dependent. The collagen density was the lowest in the high concentration Rg3-treated groups (3 and 4 mg/mL).

### Decreased expression of collagen type I

**Figure 9:**
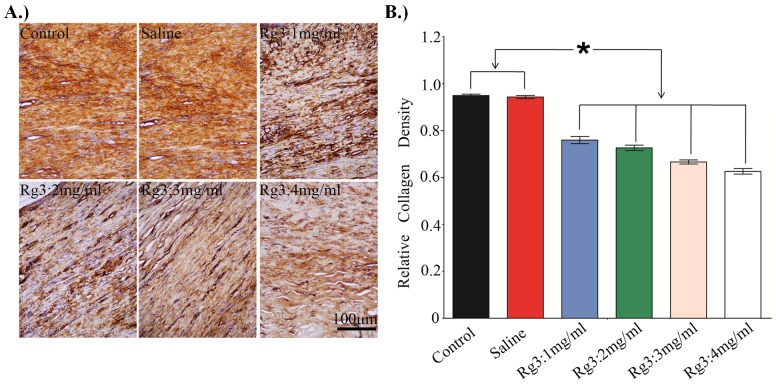
Immunohistochemical staining for Col-I on day 28. (A). Areas of collagen deposition in Immunohistochemical staining histological sections (400× magnification). (B) The relative collagen density. Data represent the mean ± SD for n = 9 (* indicates significant difference, p<0.05).

During normal wound healing, collagen synthesis is expected to be at its maximum at 1 month after wounding and decrease thereafter. Immunohistochemistry data on collagen type I expression in various groups at different time points were shown in Fig. 9. Collagen expression is lower in the Rg3 injection groups compared with the untreated group and the saline injection group, and the expression of collagen type I is the lowest in the Rg3 (4 mg/mL) injection group at 1month. Collagen type I expression is identical between the control and saline injection group. The above results indicated that the collagen produced during wound healing and HS development could be inhibited by local Rg3 injection into the wound bed.

The relative density of collagen in untreated wounds was 0.95±0.01. The collagen density was significantly reduced in wounds treated with Rg3 injection groups (0.75±0.03, 0.72±0.02, 0.67±0.02, 0.62±0.02 for 1, 2, 3, 4 mg/mL, respectively) compared with the untreated wounds and the saline injection group (0.95±0.01) at 1 month (Fig. 9). The collagen density was the lowest in the Rg3 (4 mg/mL) injection group, and the expression of collagen type I has no significance in the 3 mg/mL and 4 mg/mL injection group.

### Effect of Rg3 on the VEGF expression of the scars

**Figure 10:**
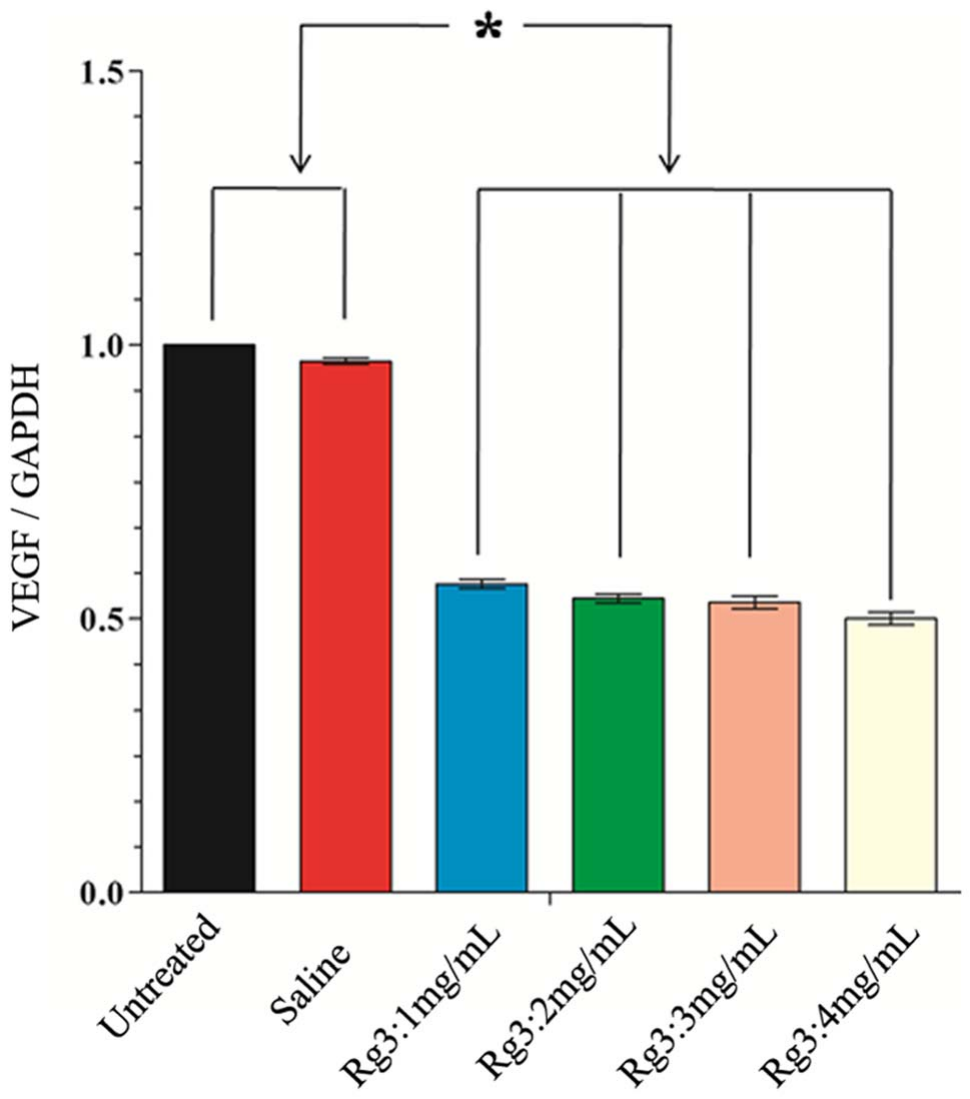
The expression of VEGF mRNA in scars on post-wounding Day 28. mRNA expressions of VEGF of the scars treated with Rg3 (1, 2, 3 and 4 mg/mL) were significantly down-regulated, in contrast to the control group and the saline-treated group. * *p<0.05*. Data represent the mean ± SD, for N = 9.

On post-wounding day 28, mRNA expression of VEGF of the HS tissues were quantified by real-time PCR. As shown in Fig. 10, VEGF mRNA expression was the highest in the control group and the saline-treated group. GAPDH was used as the internal control. To quantify the data, the ratio of VEGF/GAPDH in control group was arbitrarily set to 1.0. The ratios of VEGF/GAPDH in wounds were 0.56±0.05, 0.54±0.04, 0.51±0.05 0.50±0.06 for Rg3 concentrations of 1, 2, 3 and 4 mg/mL, respectively. These date implicated that the mRNA expressions of VEGF of the wounds treated with Rg3 (1, 2, 3 and 4 mg/mL) were significantly down-regulated, in contrast to the control group and the saline-treated group (*p<0.05*).

## Discussion

Treatment of HS is a comprehensive process. Currently, a combined treatment of intralesional injections of corticosteroids [2]–[4], interferon [5]–[9], and chemotherapeutic drugs [10], [17], [33] is the main effective and the most popular treatment for HS. However, such approaches can only be used to treat formed HS, with nothing to do with the HS forming process, thus the therapeutic results of this type of approach are not satisfactory. Furthermore, many unwelcome side effects limit further treatment. Recently published research reported that vitamin D decreases fibrosis and represents a very promising drug for antifibrogenic treatment. However, currently there has no report about its anti-scarring function on the animal yet [11]–[14]. Early intervention to inhibit HS formation, and multi-target therapy combing the therapeutic effects of corticosteroids and chemotherapeutic drug, are two promising insights to treat HS.

Rg3 has shown many biological activities, including anti-inflammatory, inhibiting tumor cell proliferation, inducing tumor cell apoptosis, and inhibiting angiogenesis [21]–[26], which makes it a very promising drug for HS treatment. Our study demonstrated that Rg3 could induce fibroblasts apoptosis, reduce exaggerated inflammation, and down regulate VEGF expression. The multi-targets therapy not only intervene the early HS formation process, but also reduce HS formation. The anti-HS results are satisfactory and no unwelcome side effects was observed. Such early intervention and combining therapeutic properties of Rg3 make it a potentially and novelty effective cure for human hypertrophic scarring.

Since regulation of fibroblast apoptosis is of great importance for controlling HS formation, and Rg3 has been shown to induce apoptosis in a variety of cell types [25], [30], [40], [41], we investigated the effects of Rg3 on HSFs in detail. The results demonstrated that Rg3 has anti-proliferative activity in HSFs. The results of CCK-8 assays showed that Rg3 could significantly inhibit HSFs cell viability in a concentration-dependent manner. HSFs treated with 100 and 200 µg/mL Rg3 displayed typical apoptotic morphology (Fig. 2). Annexin-V FITC/PI FACS analysis showed that Rg3 treatment increased the percentage of cells undergoing apoptosis in a concentration-dependent manner (Fig. 3). These results suggested that Rg3 markedly inhibited HSFs proliferation and induced apoptosis in HSFs. Our results are similar to other studies showing the pro-apoptotic effects of Rg3 on melanoma cells [42], hepatocellular cancer cells [20], colon cancer cells [40] and prostate cancer cells [35]. Therefore, Rg3 could induce apoptosis in HSFs and increase cell death.

Although inflammation is very important for healing, it is also a potent stimulator of scarring [28], [43]. It has been proposed that local wound infection can cause proliferative scar development by increasing inflammation [42]. In fact, scarless fetal wounds are characterized by a relative lack of inflammation [44]. Many reports have proved that an inhibitory effect on the early inflammatory phase of wound healing would reduce HS formation. Our study demonstrated that topical application of Rg3 significantly reduced the infiltration of inflammatory cells and controlled the excess accumulation of granulation tissue to the injured tissue at 2 week (Fig. 5). This inhibitory effect on the early inflammatory phase of wound healing had proved to have a significant outcome on the later events in the wound-healing process, namely a reduction in dermal cellularity and hypertrophy (Fig. 6).

A mounting body of evidences indicates that the hyperproliferation of fibroblasts and the excessive accumulation of collagen play an important role in HS [45]. Collagen, secreted primarily by fibroblasts, is the chief component of ECM and the principal structural protein in the dermis layer. Pathological examination showed that the number of HSFs and collagen fiber density were significantly decreased in the Rg3-treated groups, compared with the control group and the saline group. Masson's trichrome staining and collagen type I immunohistochemisty also revealed that Rg3 dramatically and dose-dependently suppressed collagen synthesis in HS tissue from the rabbit ears. Based on the *in vitro* study and the *in vivo* study, we deduced that Rg3 injection could inhibit fibroblasts proliferation, induce fibroblasts apoptosis, and thus reduce the accumulation of collagen fibers which is mainly secreted by HSFs. All these actions together indicate a reduction in HS formation.

There are considerable evidences demonstrating that an excess of microvessels present in HS, and VEGF is an important factor for angiogenesis [15]. VEGF-targeted vascular therapy is considered to be beneficial for the inhibition of angiogenesis, collagen production, and excessive scar growth in a HS [5], [46], [47]. There are many studies advocating that Rg3 can inhibit xenograft growth and angiogenesis in tumors, primarily via the down-regulation of VEGF expression [27], [40]. Thus we analyzed the expression of VEGF in HS tissues. Our study showed that VEGF mRNA expression in the Rg3-treated groups was significantly less than the control group and the saline treated group. This is in part responsible for the less red appearance of the HS observed in Rg3-treated wounds.

In summary, this study demonstrates a versatile traditional Chinese medicine; Rg3, which can alleviate HS formation in the rabbit ear model. Rg3 not only acts as an early inflammatory inhibitor and does not delay the process of wound healing, but also inhibits fibroblast proliferation, induces fibroblasts apoptosis, reduces the accumulation of collagen fibers, and down-regulates VEGF expression. Rg3 is also a safe Chinese herb, which will not cause any unwelcome side effects, compared to traditional drugs. Such early intervention and combining therapeutic properties of Rg3 make it a potential and effective cure for human HS. We propose that Rg3 can be employed to treat burn wounds, to prevent wound infection and to reduce HS development in the early phase of wound healing.
